# The nutritional impacts of soil-transmitted helminths infections among Orang Asli schoolchildren in rural Malaysia

**DOI:** 10.1186/1756-3305-5-119

**Published:** 2012-06-15

**Authors:** Abdulhamid Ahmed, Hesham M Al-Mekhlafi, Abdulelah H Al-Adhroey, Init Ithoi, Awatif M Abdulsalam, Johari Surin

**Affiliations:** 1 Department of Parasitology, Faculty of Medicine, University of Malaya, Kuala Lumpur, 50603, Malaysia; 2 Department of Parasitology, Faculty of Medicine and Health Sciences, Sana’a University, Sana’a, Yemen

**Keywords:** Soil-transmitted helminths, Anaemia, Malnutrition, Orang Asli, Malaysia

## Abstract

**Background:**

Soil-transmitted helminths (STH) infections, anaemia and malnutrition are major public health problems in school-age children in developing countries. This study was conducted on 289 Orang Asli (aboriginal) schoolchildren in order to assess the current prevalence and predictors of anaemia and malnutrition, as well as the nutritional impacts of STH infections among these children.

**Methods:**

A cross-sectional study was combined with a longitudinal follow-up three months after treatment with anthelminthic drugs. Blood samples were collected from the children to measure haemoglobin (Hb) level. Anthropometric and socioeconomic data were also collected and the children were screened for STH.

**Results:**

The baseline findings revealed that the prevalence of anaemia, significant stunting, underweight and wasting among the children were 41.0%, 28.0%, 29.2% and 12.5%, respectively. Overall, the prevalence of trichuriasis, ascariasis and hookworm infections were 84.6%, 47.6% and 3.9%, respectively. Haemoglobin level was significantly lower among the moderate-to-heavy infected children compared to the negative-to-light infected children. Age <10years and moderate-to-heavy ascariasis were the predictors of anaemia. Stunting was associated with gender, age, moderate-to-heavy ascariasis and trichuriasis. Three months post-treatment assessment showed that the moderate-to-heavy infected children gained significant increment in their mean Hb level compared to the negative-to-light infected children (0.44 g/dL compared to 0.08 g/dL). However, no difference was found in the mean increments in growth indices between the groups.

**Conclusion:**

STH infections, anaemia and malnutrition are still prevalent and a matter of public health concern in Orang Asli communities in Malaysia. Sustainable deworming programme at school and community levels among these populations will help to improve their health and nutritional status.

## Background

Human beings dwelling in poor and disadvantaged communities with inadequate hygiene in the developing countries of the world continue to harbour and endure the burden of soil-transmitted helminths (STH) infections. The disease burden is mainly manifested as nutritional stress and associated with poor appetite, food indigestion and malabsorption, impaired growth and anaemia [1-3]. Anaemia, malnutrition and STH infections are prevalent throughout the developing nations of the world. They often occur synergistically in areas of low socioeconomic status, where they constitute a major public health problem especially among children of school age [4]. The highest prevalence of malnutrition in the world occurs in Asia where about 70% of all the children are malnourished [5].

An estimated 30% of the world’s total population are anaemic [6]. In East Asia, nearly 50% of all the school age children are anaemic [7]. The burden of STH infections is associated with anaemia and micronutrient deficiencies such as iron, vitamins and folate. This leads to reduced work capacity [8], poor cognitive function [9] and pregnancy disorders [10]. Anaemia and malnutrition increase the risk and severity of infections among the affected individuals and hence, are major causes of death especially among children and pregnant women [7,11]. The synergistic occurrence of helminthiasis, anaemia and malnutrition exert a negative effect on growth and development of the affected person [12].

In Malaysia, STH infections, anaemia and malnutrition are still prevalent and of public health concern especially among the low income groups living in rural areas. For instance, according to the Malaysian Ministry of Health [13], 18.3% and 20.8% of pre-school girls and boys in the country were anaemic. Previous research studies among the low income groups reported prevalence rates of anaemia ranging from 24.4% - 48.5% [14-17], as well as varying degrees of malnutrition. The Orang Asli (aboriginal) population of Malaysia are known to be lagging behind in terms of educational attainment, health care and general socio-economic status. They are also known to harbour a range of parasitic diseases including STH infections [18-20]. Hence, this study was carried out to determine the current prevalence of anaemia and malnutrition and to assess the effects of STH infections on the nutritional status of Orang Asli schoolchildren in an aboriginal setting in rural Malaysia.

## Methods

### Study area and subjects

The study was conducted in Satak area, Raub district, Pahang state, located about 200 kilometres northeast of Kuala Lumpur, between latitude 3°59’22”N - 4°02’03”N and longitude 101°37’48”E - 101°43’47”E. The area comprise of five indigenous communities namely; Pos Satak, Sungai Kelang, Sungai Rensong, Ruai Hulu and Ruai Hilir. These communities are the catchment centres for the Sekolah Kebangsaan Satak (National school of Satak).

The study subjects include all the volunteered, apparently healthy children enrolled in the school at the time of the study. The school has a total enrolment of 364 pupils. However, two hundred and eighty nine (289) eligible pupils aged 6 – 13 years were available during our visits to the school and they voluntarily participated in this research. Among them, 254 children delivered stool samples for examination. Despite the high response rate (88% response rate), few aspects should be considered when conducting research among Orang Asli schoolchildren. First, risk of selection bias is present if children in high numbers are absent as the cause of the absenteeism can be related to the health problem under investigation by the research. Second, the high absenteeism rate reported among these children may result in high dropout rate and subsequently affect the power of longitudinal studies. During our visits for post-treatment assessment, few students were absent. However, we managed to collect their blood and stool samples, and anthropometric measurements in their home settings.

### Study design

This was a cross-sectional study with a longitudinal follow-up (pre and post-treatment assessment). Anthropometric data, stool and blood samples were collected from the subjects at baseline (pre-treatment) and at follow-up (three months post-treatment). Due to ethical reasons we could not use a placebo control group. However, data were compared between the children who had moderate-to-heavy infections with STH and those who were un-infected or had only light infections (negative-to-light). It is well documented that moderate-to-heavy STH infections are clinically significant and are associated with a wide range of symptoms, whereas light infections are usually asymptomatic. Moreover, stunting which represents a state of chronic nutritional stress is often seen as the best indicator of malnutrition [21]. Hence, in this study, nutritional differences between the groups of the children were calculated with reference to the prevalence of significant stunting.

### Questionnaire

Socioeconomic and demographic data of the participants were collected using a pre-tested questionnaire. Several visits were paid to the villages, where the children and their parents were interviewed in order to gather information on their socioeconomic status, personal hygiene practices and medical history.

### Haemoglobin assessment

Haemoglobin (Hb) level of each child was measured on the spot by trained nurses. A finger pricks blood was obtained from each child and assessed directly by using the Hemocue hemoglobinometer (Hemo Cue, AB, Angelhom, Sweden). All children with Hb level <12g/dL were considered to be anaemic [6].

### Anthropometric assessment

Height and weight of all the subjects were measured and recorded separately by two researchers to avoid intra-human errors, and the average measurements for each child were used for the data analysis. The children were measured wearing light uniforms, without shoes, belts, caps or any other material that could tamper with their actual heights and weights. Weight was measured to the nearest 0.1kg using a SECA Scale. Height was recorded to the nearest 0.1cm, using the same device which has a vertical scale and a head piece attached to it for height measurement.

### Parasitology

Fresh stool samples were collected from 254 subjects in clearly labelled containers with wide mouth and screw cap. Appropriate demonstrations on how to collect the stool samples were conducted to them to avoid possible contamination in the process of collection at home. The collected stool samples were transported immediately in suitable ice boxes for analysis at the stool processing laboratory of the department of Parasitology, University of Malaya. The samples were analyzed using the Kato-Katz and Harada Mori Techniques [22]. Egg counts were read accordingly and recorded as eggs per gram of faeces (epg). Intensity of infection was determined and recorded as light, moderate or heavy according to the WHO guidelines [23]. Duplicate Kato-Katz slides were prepared from each stool specimen and all were examined twice by two different microscopists and the average readings were used to make this report.

### Anthelminthic treatment

All the infected children were treated with a 3-day course of 400 mg albendazole tablets, Zentel® (GlaxoSmithKline, London, UK). The drugs were administered by a team of medical personnel, the researchers and the school’s deputy headmaster in a direct observed therapy. The orange flavour of the drugs encouraged the children to chew the tablets before swallowing. Packets of fruit juices were also given to them to drink along with the tablets. About 14 days after deworming, stool samples were collected again from the pupils and examined in order to ascertain the efficacy of the drug. Light infections were still detected in 20 (8.4%) children (16 with trichuriasis and 4 with ascariasis), and were treated accordingly with a single dose of 400 mg albendazole to ensure complete deworming.

### Statistical analysis

The anthropometric data of the children was analyzed by using the Epi-Nut component of the EpiInfo software (version 3.4.3, 2007, Centre for Disease Control and Prevention, Atlanta, Georgia, USA), to obtain the relevant Z-Scores for Height-for-Age (HAZ), Weight-for-Age (WAZ) and Weight-for-Height (WHZ). The values were assessed with reference to the National Centre for Health Statistics (NCHS) and the WHO values. Children who had Z-Scores < -2SD of the NCHS reference values were considered as significantly malnourished. Data analysis was performed by using the SPSS software version 13.0 for windows (SPSS, Chicago, IL. USA). The distribution of quantitative data was examined using the Shapiro-Wilk test and found to be normal. The level of significance of 0.05 was used for all the statistical tests. Univariate analysis was used to assess the risk factors of anaemia and significant stunting among the subjects. However, variables that showed association with a *P*-value ≤ 0.20 were used to develop a stepwise forward multiple regression model, in order to retain all possible significant associations of related variables with anaemia and stunting [24]. Independent t-test was used to examine the differences and improvements in Hb, weight and height between groups. Repeated measures analysis of variance (ANOVA) was used to compare the Hb, weight and height at pre-treatment and post-treatment assessments, after adjusting for the age and sex of the participants. Moreover, analysis of covariance (ANCOVA) was used to adjust for the effect of the pre-treatment Hb level as a covariate.

### Ethical consideration

The protocol of this study was reviewed and approved by the Medical Ethics Committee of the University of Malaya Medical Centre, Kuala Lumpur (Reference Number: 788.74). Oral informed consent was duly obtained from all the study subjects, their parents and the school authority before embarking on the survey. Written permission was also obtained from the education authorities in Raub district. The objectives and protocols of the research were thoroughly discussed with the community leaders and school management for proper clarification. It was agreed that, participation in the research is voluntary and that subjects may withdraw from the research at any time without prior notice.

## Results

A total of two hundred and eighty nine pupils (140 males and 149 females), aged 6 – 13 years old with a mean age of 9.7 ± 0.2 years participated in this study. About half of the parents had no formal education and almost two thirds of the children belong to families with low monthly income (<RM500.00/month; 1US$ = RM3.00). Almost half (53%) of the children were ≥10 years old and about one fifth of all the children belong to large family settings (>7 members). About half of the households in the study area have no toilet facilities, rivers were used as the preferred defaecation sites as well sources of water for domestic usage. Only about one fifth of the households in the area have access to piped water supply. The results of this study showed that 93.7% of all the participants were infected with at least one species of STH.

The prevalence of trichuriasis, ascariasis and hookworm infections were 84.6%, 47.6% and 3.9%, respectively. Among those, about a quarter of the infections by *Ascaris lumbricoides* and nearly half of those by *Trichuris trichiura* were of moderate-to-heavy intensities, whereas, all hookworm infections were of light intensity. Overall, the combined moderate-to-heavy STH infections accounted for 60.5% of all the infections. Data on the STH prevalence and distribution among these children have been published previously [18].

At baseline, the mean haemoglobin levels of the participants was 12.2 ± 0.14 g/dL, and 96 (41.0%) of the subjects were found to be anaemic. Moreover, the mean weights and heights of the study subjects were 24.2 ± 0.44 kg and 126.8 ± 0.61 cm, respectively. The prevalence of significant stunting, underweight and wasting was 28.0%, 29.2% and 12.5%, respectively. The baseline anthropometric characteristics of the moderate-to-heavy STH infected and the negative-to-light infected children showed that there is no significant difference (*P* > 0.05) in terms of weight and height among them. However, the children who had moderate-to-heavy STH infections had significantly lower mean haemoglobin concentration compared to those who had negative-to-light infections (11.9 g/dL compared to 12.6 g/dL; *P* = 0.005). Although the children who had moderate-to-heavy infections were more anaemic compared to the negative-to-light infected group, the difference was not statistically significant (*P* = 0.099). The general baseline anthropometric and nutritional characteristics of the two groups of subjects are shown in Table 1.

**Table 1:** Nutritional characteristics of the participants according to STH infections (n = 254)

**Variables**	**All subjects (n = 254)**	**STH infections**		
		**Negative-to-light (n =110)**	**Moderate-to-heavy (n = 144)**	***P***
***Before treatment (Baseline)***				
Haemoglobin (g/dL)*	12.2 ± 0.14	12.6 ± 0.14	11.9 ± 0.13	0.005‡
Weight (kg)*	24.2 ± 0.44	23.9 ± 0.42	24.4 ± 0.54	0.607
Height (cm)*	126.8 ± 0.61	125.9 ± 0.95	127.5 ± 0.75	0.198
Anaemia†	96 (40.2)	34 (34.0)	62 (44.6)	0.099
Significant stunting†	63 (24.8)	18 (16.4)	45 (31.3)	0.006‡
Significant underweight†	68 (26.9)	30 (27.3)	38 (26.6)	0.901
Significant wasting†	35 (13.8)	20 (18.2)	15 (10.4)	0.075
***Improvements after treatment***				
Haemoglobin (g/dL)*	0.29 ± 0.07	0.09 ± 0.04	0.44 ± 0.12	0.048‡
Weight (kg)*	1.16 ± 0.06	1.10 ± 0.07	1.20 ± 0.08	0.459
Height (cm)*	1.22 ± 0.07	1.30 ± 0.09	1.15 ± 0.09	0.288

*Values are mean ± SEM; Independent samples t-test.

†Values are number (%); Chi square test.

‡Significant difference between groups.

Examining the association of anaemia with the possible risk factors revealed that age <10 years (χ^2^ = 4.817; *P* = 0.028) and moderate-to-heavy ascariasis (χ^2^ = 3.867*; P* = 0.049) were significantly associated with anaemia (Table 2). Multivariate analysis using logistic regression confirmed that age <10 years (OR = 2.11; 95% CI = 1.23, 3.63; *P* = 0.007) and moderate-to-heavy ascariasis (OR = 2.05; 95% CI = 1.12, 3.76; *P* = 0.021) as the main predictors of anaemia among the study subjects (Table 3).

**Table 2:** Univariate analysis for the potential risk factors of anaemia among Orang Asli schoolchildren in Satak, Pahang (n = 254)

**Variables**	**Anaemia**		***P***
	**Prevalence (%)**	**OR (95% CI)**	
**Gender**			
Male	45.1	1.39 (0.86, 2.26)	0.181
Female	37.1	1	
**Age group**			
<10 years	48.0	1.72 (1.06, 2.80)	0.028*
≥10 years	34.9		
**Mother’s educational level**			
No formal education	44.9	1.42 (0.87, 2.31)	0.160
≥ 6 years formal education	36.5	1	
**Mother’s employment status**			
Not working	41.5	1.42 (0.47, 4.27)	0.533
Working	33.3		
**Family Size**			
>7 members	43.1	1.12 (0.62, 2.00)	0.717
≤7 members	40.5	1	
**Household monthly income**			
<RM500.00	44.6	1.48 (0.90, 2.45)	0.124
≥RM500.00	35.2	1	
***Ascaris*infection**			
Moderate-to-heavy	50.8	1.80 (1.00, 3,23)	0.049*
Negative-to-light	36.5	1	
***Trichuris*infection**			
Moderate-to-heavy	41.0	1.07 (0.64, 1.80)	0.793
Negative-to-light	39.0	1	
**Hookworm infection**			
Positive	55.6	1.91 (0.50, 7.30)	0.337
Negative	39.6	1	
**Stunting**			
Significant	40.8	1.03 (0.57, 1.87)	0.914
Normal/mild	39.8	1	
**Underweight**			
Significant	45.2	1.34 (0.75, 2.41)	0.327
Normal/mild	38.1	1	
**Wasting**			
Significant	42.9	1.09 (0.50, 2.20)	0.813
Normal/mild	40.8	1	

*Significant association (*P* < 0.05).

**Table 3:** Multivariate analysis for the potential risk factors of anaemia among Orang Asli schoolchildren in Satak, Pahang (n = 254)

**Variables**	**Anaemia**	***P***
	**OR (95% CI)**	
Age (<10 years)	2.11 (1.23, 3.63)	0.007*
Gender (male)	1.49 (0.87, 2.57)	0.149
*Ascaris* infections (Moderate-to-heavy)	2.05 (1.12, 3.76)	0.021*
Mother’s educational level (No formal education)	1.37 (0.78, 2.41)	0.270
Household monthly income (<RM500)	1.35 (0.75, 2.42)	0.312

*OR*: odds ratio; *CI*; Confidence interval.

*Significant risk factor (*P* < 0.05).

Variables entered in the model were age, moderate-to-heavy ascariasis, gender, mother’s educational level and household monthly income.

The association of stunting with socioeconomic and health factors among the children that participated in this study was examined and the result showed that, gender (male) (χ^2^ = 4.137; *P* = 0.042), age ≥ 10 years (χ^2^ = 32.86; *P* < 0.001), moderate-to-heavy ascariasis (χ^2^ = 5.687; *P* = 0.017) and moderate-heavy trichuriasis (χ^2^ = 5.069; *P* = 0.024) were significantly associated with stunting (Table 4). Logistic regression analysis confirmed age ≥ 10 years (OR = 5.06; 95% CI = 2.60, 9.87; *P* < 0.001), male gender (OR = 2.82; 95%CI = 1.48, 5.39; *P* = 0.002) and moderate-to-heavy ascariasis (OR = 2.17; 95% CI = 1.10, 4.26; *P* = 0.025) as the main predictors of stunting among these children (Table 5).

**Table 4:** Univariate analysis for the potential risk factors of stunting among Orang Asli schoolchildren in Satak, Pahang (n = 254)

**Variables**	**Significant stunting**		***P***
	**Prevalence (%)**	**OR (95% CI)**	
**Gender**			
Male	33.6	1.71 (1.02, 2.87)	0.042*
Female	22.8	1	
**Age group**			
≥10 years	42.2	2.90 (1.84, 4.56)	<0.001*
<10 years	11.9	1	
**Mother’s educational level**			
No formal education	29.2	1.14 (0.68, 1.90)	0.630
≥6 years formal education	26.7	1	
Not working	28.5	1.59 (0.45, 5.38)	0.477
Working	20.0	1	
**Family size**			
>7 members	32.8	1.34 (0.74, 2.45)	0.334
≤7 members	26.7	1	
**Household monthly income**			
<RM500	27.1	0.89 (0.53, 1.51)	0.665
≥RM500	29.5	1	
***Ascaris*infection**			
Moderate-to-heavy	35.9	2.10 (1.13, 3.90)	0.017*
Negative-to-light	21.1	1	
***Trichuris*infection**			
Moderate-to-heavy	31.0	1.94 (1.09, 3.48)	0.024*
Negative-to-light	18.8	1	
**Hookworm infection**			
Positive	10.0	0.33 (0.04, 2.63)	0.269
Negative	25.4	1	
**Anaemia**			
Anaemic	26.8	0.95 (0.77, 1.18)	0.664
Normal	29.2	1	

*Significant association (*P* < 0.05).

**Table 5:** Multivariate analysis for the potential risk factors of stunting among Orang Asli schoolchildren in Satak, Pahang (n = 254)

**Variable**	**Significant stunting**	***P***
	**OR (95% CI)**	
Age (>10 years)	5.06 (2.60, 9.87)	<0.001*
Gender (Male)	2.82 (1.48, 5.39)	0.002*
*Ascaris* infections (Moderate-to-heavy)	2.17 (1.10, 4.26)	0.025*
*Trichuris* infections (Moderate-to-heavy)	1.36 (0.71, 2.60)	0.358

*OR*: odds ratio; *CI*; Confidence interval.

*Significant risk factor (*P* < 0.05).

Variables entered in the model were age, gender, moderate-to-heavy ascariasis and moderate-to-heavy trichuriasis.

**Figure 1:**
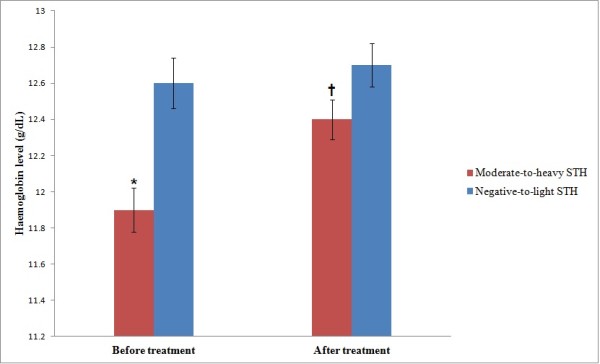
Haemoglobin level in relation to soil-transmitted helmiths infections among the Orang Asli schoolchildren in Satak, Pahang (n = 254). Values are mean ± SEM.^*^Significant difference (lower) compared to negative-to-light STH infection group (Independent t-test, *P* < 0.05). †Significant difference (higher) compared to before treatment (repeated measures ANOVA, *P* < 0.05).

Three months after the administration of anthelminthic treatment to all the infected children, the mean increments in Hb and anthropometric indices between the negative-to-light and the moderate-to-heavy infected groups were assessed (Table 1). The result showed that the mean increments in Hb levels was significantly higher among moderate-to-heavy infected group as compared to the negative-to-light infected group (0.44 g/dL compared to 0.09 g/dL; *t* = -1.987; *P* = 0.048). The repeated measures ANOVA showed that the Hb level increased significantly (F = 9.243; *P* = 0.003) among the moderate-to-heavy STH group (12.4 g/dL vs 11.9 g/dL) as compared to the negative-to-light infected group (12.5 g/dL vs 12.6 g/dL) (Figure 1). After adjusting for the effects of age and gender of participants (using repeated measures ANOVA), and the pre-treatment Hb level (using ANCOVA), the difference in Hb increments between the two groups remained significant. On the other hand, the results of repeated measures ANOVA showed no significant difference in the mean increments of weight and height between the two groups (*P* > 0.05).

## Discussion

To the best of our knowledge, this is the first cross-sectional study with a longitudinal follow-up that attempts to investigate the impact of STH infections on the nutritional status of Orang Asli children in Malaysia as well as the possible benefit of deworming on the affected individuals. STH infections, anaemia and malnutrition remain among the major public health problems afflicting underprivileged people, particularly in developing countries [4]. Children from rural communities tend to be more vulnerable due to their low socioeconomic status and social isolation [25]. It is estimated that Asia had the highest number of malnourished children in the world [5].

The findings of our study indicated that 41.0% of all the subjects examined were anaemic. This is in agreement with the results obtained in previous studies among aboriginal children in Malaysia [14,15]. A similar prevalence was reported elsewhere [26,27]. However, lower prevalence rate was reported among the Malaysian adults [17]. The high prevalence reported in this study could be related to the fact that the study population live in remote rural communities with high rate of poverty which contribute to poor access to good diet and proper healthcare.

The results of this study also showed that malnutrition is common among the study subjects. The prevalence of significant stunting, underweight and wasting were; 28.0%, 29.2% and 12.5%, respectively. Our findings were consistent with the results of some previous reports among some Malaysian populations [19,28,29]. Higher prevalence of malnutrition was also reported in India [30], Nigeria [31], and Brazil [32]. The high prevalence of malnutrition by our study likely reflects the low socioeconomic status of most of the households in Orang Asli communities. Low socioeconomic status relates to poverty which affects the purchasing power of an individual or group, hence affecting the dietary intake. The long established synergism with helminthiasis may also contribute to the high prevalence of malnutrition among these subjects.

Our study showed that age < 10 years was a significant risk factor of anaemia among these children. This is consistent with the findings by Calis *et al.,*[33] who reported that the Malawian pre-school and early school-age children had the highest risk of anaemia. It is also in agreement with the findings of a previous study among Orang Asli children by Al-Mekhlafi *et al.,*[14] which demonstrated that the prevalence of anaemia decreased significantly with age. It is well known that females tend to be more anaemic than males especially in the reproductive age as a result of physiological differences. However in the present study no significant difference in the prevalence of anaemia was found between males and females. Previous studies from Kenya [34] and Tanzania [35] demonstrated high prevalence of anaemia among the male compared to the female schoolchildren.

Moderate-to-heavy ascariasis was also found to be significantly associated with anaemia among these subjects. The association between anaemia and intestinal helminthiasis has been reported previously [15,31]. However, in a previous study among Malaysian adults [17], no association was found between anaemia and helminth infections, probably due to the very low prevalence of helminth infections. It was suggested that blood loss due to hookworm and *T. trichiura* infections strongly correlated with worm load [36]. Moreover, it was reported that even light hookworm infections could be associated with lower haemoglobin concentration and higher prevalence of anaemia [34]. Although ascariasis is known to influence the nutritional status, its impact on anaemia is less clear [37]. The strong association between anaemia and moderate-to-heavy ascariasis observed among these children could be attributed to the heavy intensity of infections and the long synergism with other contributory factors such as poverty and poor dietary intake. As a limitation, the present study did not measure the daily iron intake. However, a previous study had shown that the daily iron intake by Orang Asli children in rural Malaysia was only about 29% to 49% of the recommended daily intake [14].

Our investigation revealed that children below the ages of 10 years old were significantly less stunted compared to those ≥10 years. This is in agreement with previous similar studies [38-40]. It has been established that stunted children continue to deviate from normal growth with increasing age. Hence, the risk of becoming stunted continues as children get older. In this study, male children tend to be significantly more stunted than the females. Male sex, have been reported as a risk factor of stunting in a previous study in Indonesia [40]. Conversely, in a study among under-five children in Nepal, girls were reported to be at higher odds for stunting [41]. We assume that the higher odds of stunting among the boys in our study could be attributed to limited food supply in many households and coupled with the fact that the boys are more likely to suffer from hard tasks at home such as farming, fishing and other domestic upkeep activities.

Our study also demonstrated that moderate-to-heavy intensities of infection with *T. trichiura* or *A. lumbricoides* are significant predictors of stunting among the children. This is consistent with the findings from previous studies in Peru [38], China [42] and Tanzania [43]. Trichuriasis and ascariasis tend to be very prevalent among children of school age in many endemic areas. More so, STH infections are associated with decreased appetite and low food intake [3], which result to decreased growth rate, poor fitness, decreased activity and poor cognitive function. Micronutrient losses and nutrient malabsorption due to ascariasis and blood-loss due to trichuriasis can lead to iron deficiency, iron deficiency anaemia and poor growth rate [3,44].

Post-treatment assessments following anthelminthic treatment showed that the moderate-to-heavy infected children benefitted more from the deworming and gained more weight than the negative-to-light infected children but the difference was not statistically significant. This agrees with previous studies conducted in India [2], Jamaica [45] and Guatemala [46]. However, Stephenson *et al.,*[3] reported significant improvements in height and weight among the treated Kenyan school boys harbouring multiple helminth infections at 4 months after treatment.

In the present study, the moderate-to-heavy infected children showed a significant increment in the mean haemoglobin concentration after 3 months compared to the negative-to-light infected children. This is in agreement with a clinical trial conducted among Zanzibari preschool children which reported that anthelminthic treatment using mebendazole, significantly reduces anaemia status among the children [47]. The improvement in haemoglobin levels among the subjects following anthelminthic treatment lead to the assumption that the burden of helminthiases is a significant contributor to anaemia in the population.

## Conclusions

Although meaningful achievements have been recorded all over Malaysia in terms of quality of life improvement throughout the years of independence, the Orang Asli (aboriginal) population seems to be left behind by other races in the country such as the Malays, Chinese and Indians, probably due to their remoteness and the desire to stay in the jungles and away from other people. Previous reports findings have shown that the Orang Asli all over the country share similar socioeconomic, health and demographic characteristics. Although their number constitute about 0.6% of the country’s total population, their plight is of great concern. High prevalence of STH infections, anaemia and malnutrition evidently persist among Orang Asli children resulting in negative consequences on their growth, health and overall productivity. In our survey, anthelminthic treatment has produced some positive changes on the nutritional status of the more heavily infected children. It is therefore suggested that beside poverty alleviation and health education, helminth control should be considered as an important intervention programme for the Orang Asli population, both at school and community levels.

## Competing interests

The authors’ declare that they have no competing interests.

## Authors’ contributions

AA was involved in all phases of the study, including study design, data collection, data analysis and write up of the manuscript; JS, HMA and II supervised the study, and revised the manuscript; HMA was involved in the statistical analysis of data; AHA and AMA were involved in the collection and laboratory examination of samples. All authors read and approved the final manuscript. JS and HMA were the guarantors of the paper.
